# Healthcare Workers’ Perception of Measures to Reduce the Risk of New Tuberculosis Infections: A Qualitative Study Report

**DOI:** 10.3390/nursrep12040084

**Published:** 2022-11-17

**Authors:** Debra Madzinga, Takalani Grace Tshitangano, Ndidzulafhi Selina Raliphaswa, Lufuno Razwiedani

**Affiliations:** 1 Department of Public Health, University of Venda, Thohoyandou 0950, South Africa; 2 Department of Advanced Nursing Science, University of Venda, Thohoyandou 0950, South Africa; 3 Department of Public Health Medicine, Sefako Mkgatho Health Sciences University, Ga-Rankuwa 0221, South Africa

**Keywords:** healthcare workers, infections, new, tuberculosis, perception, implementation, Collins Chabane Municipality, South Africa

## Abstract

Tuberculosis, which is an infectious airborne disease remained the main leading cause of death in South Africa for three consecutive years from 2016 to 2018. In 2020 alone, the country had an estimated 328,000 people who suffered from TB, with 61,000 dying from it. Collins Chabane Municipality had 129 and 192 new TB cases recorded in 2017 and 2018, respectively, which is far from reaching the END TB STRATEGY targets. WHO scientific evidence demonstrates that TB control measures are effective in reducing the spread and development of new cases. Though scientific evidence revealed negative attitudes towards the recommended TB control measures in public hospitals of the Vhembe district, a deeper understanding of these attitudes is needed to remedy the situation. This study aimed to describe healthcare workers’ perceptions of TB control measures at Collins Chabane Municipality in South Africa. A qualitative, exploratory case study design was adopted. Multi-stage sampling technique was used to select both the healthcare facilities and the participants. Only 24 healthcare workers trained on tuberculosis management were voluntarily recruited. However, data were saturated at the twelfth (12) participant purposively selected from six healthcare facilities of Collins Chabane Municipality. Data collected through unstructured in-depth individual interviews were analyzed thematically. The proposal for this study was ethically cleared by the University of Venda Ethics Committee (SHS/20/PDC/35/1111). Results indicate that TB administrative, environmental and respiratory control measures are well understood by health workers even though there are challenges with implementation concerning some, such as closing windows during winter, UVGI lights that are non-functional and taking too long to be fixed, no specimen collection during weekends and holidays thereby delaying TB diagnosis and lack of skills concerning how to use respirators and cough etiquette. The Vhembe district TB control programme should intensify infection control training and continue monitoring giving the needed support.

## 1. Introduction

Tuberculosis (TB) remained the main leading cause of death in South Africa for three consecutive years from 2016 to 2018 [1]. It is an airborne disease, which is caused by the bacillus *mycobacterium tuberculosis*. In most cases, it affects the lungs, but it can also affect other parts of the body (termed extra pulmonary TB). It is transmitted from one person to another when a person infected coughs, sneezes or spits, propelling *mycobacterium tuberculosis* into the air [2].

This infectious disease remains a global public health concern too. In 2019, the number of new TB cases was 10.0 million [1,3]. Most of the cases that occurred in 2017 were in the WHO South East Region at 44%, the WHO African Region at 25% and the WHO Western Pacific Region at 18% [4]. A smaller proportion of new TB cases occurred in the WHO Eastern Mediterranean Region at 7.7%. the WHO Region of the Americas at 2.8% and the WHO European Region at 2.7% [4]. Since 2017, the European Region has had a decline in TB incidence rate of 4.75 per year. The TB mortality rate also fell at a rate of 59% in the region. Africa is one of the regions with the highest burden of TB. In 2017 alone, the African continent had about 237 cases per 100,000 people. The continent had 12 million people who died from TB making it the slowest in reducing the mortality rate.

In 2020, South Africa was one of the 30 highest TB burdened countries and one of the 8 countries that account for two thirds of the new TB cases globally [4]. According to Chakaya et al. [5], around 322,000 people fell ill with TB in South Africa in 2017, though there was a decline in the number of incidences when compared to the year 2011. About 80% of the population aged 30 to 39 years had latent TB in South Africa [2]. However, funding falls far short of what is needed in low- and middle-income countries (LMICs), with spending in 2020 amounting to USD 5.3 billion, less than half (41%) of the global target [4]. Furthermore, data revealed that funding in 2021 remained inadequate despite the increase in both domestic and international funding for TB [5].

South Africa introduced the GeneXpert testing machine, which diagnoses TB and issues results within a few hours, making it easy to detect TB earlier and therefore initiate treatment promptly. This initiative assists in reducing TB infection transmission because if people are given treatment earlier, there is a higher chance they will not transmit the bacteria to other people. In addition, HIV-positive individuals are to be started on Isoniazid Preventive Therapy (IPT) for a period of 6–9 months to prevent the development of TB disease [4,6].

In response to the TB burden, South Africa developed the National Tuberculosis Guideline in 2014, with the aim of reducing TB incidences, since TB is one of the drivers of the morbidity and mortality rate in South Africa. The guideline was developed to guide healthcare workers who are managing TB patients on how to screen patients for TB, initiate treatment for all types of TB, keep patients on treatment until they have completed their treatment and prevent TB amongst people who are living with HIV/AIDS. In addition, the South African National Infection Prevention and Control Policy & Strategy was developed in 2007 [7], as a guide to the South African healthcare system, in a joint effort to prevent and control infectious diseases in healthcare systems. This was replaced by the 2020 South African Nation TB infection control policy and strategic framework [8], which included TB infection control measures such as administrative, environmental and respiratory controls, as recommended by the World Health Organization.

According to the WHO [8], scientific evidence reveals that triage (which is one of the administrative controls) has an absolute risk reduction of 6% for latent TB Infection (LTBI) incidence among health workers in all settings (*n =* 6); and when disaggregating by burden of disease, a 3% absolute risk reduction in LTBI was observed among health workers in low TB burden settings (*n =* 5), compared with a 1.7% reduction in high TB burden settings (*n =* 1). In addition, there is a 12.6% absolute risk reduction in the number of active TB disease cases in persons attending healthcare settings with the use of triage (in combination with other IPC measures) when compared with similar populations in settings where triage was not implemented.

With regards to respiratory isolation (which is one of the respiratory controls), scientific evidence contained in [9] reveals that there is an absolute risk reduction of 2% in health workers when persons with presumed TB and confirmed TB patients underwent respiratory separation or isolation; and when data were disaggregated by TB burden (low versus high), there was a relatively small reduction in risk of acquiring LTBI when respiratory separation or isolation was implemented. However, no significant differences in absolute risk reductions were observed between low and high TB burden settings (1.6% versus 1.9%). Thus, the use of surgical masks by people with presumed or confirmed TB was associated with 14.8% risk reduction in incidences of TB infection among health workers. The use of mechanical ventilation in combination with other environmental controls (such as ultraviolet germicidal irradiation (UVGI) lights) was associated with a 4.1% reduction in TST conversion among healthcare staff [8].

Regardless of effectiveness knowledge concerning the recommended administrative, environmental and respiratory TB infection control measures, the number of new TB cases increased in Collins Chabane Municipality, with 129 and 192 new TB cases recorded in 2017 and 2018, respectively [10]. These numbers show that the municipality is far from reaching the END TB STRATEGY targets of reducing the mortality rate by 90% and incidence rate by 80% by 2030 [8]. Many reasons for not reaching the TB targets were revealed by previous studies. Reference [11] found that rural public hospitals in the Vhembe district where Collins Chabane Municipality is based implemented ineffective tuberculosis control measures. Although [12] revealed negative attitudes towards recommended administrative, environmental and respiratory TB infection control measures as one of the factors contributing to the implementation of ineffective TB infection control measures in public hospitals of the Vhembe district, a deeper understanding of these negative attitudes is needed if interventions to remedy the situation are to be initiated. This study described the perceptions of healthcare workers regarding TB administrative, environmental and respiratory control measures at Collins Chabane Municipality and highlighted the gaps that still needed the district TB control teams’ attention.

## 2. Materials and Methods

### 2.1. Study Design

A qualitative phenomenological design underpinned this study to explore healthcare workers’ perceptions of TB administrative, environmental and respiratory control measures. This design was chosen to provide rich and full information as lived and experienced by healthcare workers. The Consolidated Criteria for Reporting Qualitative studies (COREQ) [13] was used to create the section below demonstrating how this paper addresses issues of rigor (how participants were engaged); representation (whether the right participants were engaged); and reflexivity (whether engagement was carried out in ways that are meaningful, ethical and equitable).

### 2.2. Setting

The study was conducted in Collins Chabane Municipality of the Vhembe district. The Vhembe district is located in deep rural areas of the Limpopo Province, South Africa, and has four municipalities, namely Collins Chabane Municipality, Thulamela, Makhado and Musina. To the northeast, Collins Chabane Municipality’s borders extend to Mozambique and on the southeast to Kruger National Park. A total of 347,975 people reside in the municipal area of 50,003 km^2^. Africans (347,109) made up the largest number of people staying in Collins Chabane Local Municipal area, followed by Indian/Asian (301), Coloureds (294) and Whites (271). In this municipality, the majority of healthcare workers in public healthcare facilities are African females.

Data were collected at six of the 20 clinics with the highest number of new TB cases around Collins Chabane Municipality. Interviews were conducted in a quiet room occupied by the first author and one participant at a time. The non-probability purposive sampling method was used to select samples from both the healthcare workers and healthcare facilities. Twenty-four (24) healthcare workers trained on TB management (aged between 23 and 54 years; 8 females and 8 males; 13 professional and 3 staff nurses; 11 with nursing degree and 5 with nursing diplomas; between 2 and 24 years of nursing experience) were recruited face to face from the selected healthcare facilities to participate in the study. However, sample size was dependent on data saturation. None of the recruited participants refused or dropped out until the end of the study. Table 1 below has the details:

### 2.3. Data Collection

The first author (who is a female qualified registered nurse with five years of primary healthcare nursing experience and an MPH student on research training) conducted the interviews. A relationship was built to ease the interaction with the recruited participants. Data were collected by means of unstructured in-depth individual interviews from the participants who indicated their willingness to participate in the study by signing consent forms. This was done after information about the researcher’s characteristics, personal goals and interest in the topic as well as the purposes for conducting the research study were given to the participants. Ethical clearance was obtained from the Ethics Committee of the University of Venda (SHS/20/PDC/35/1111). Data were collected after permission was granted to access the healthcare facility and to conduct the study by the Provincial Department of Health, the Vhembe district executive manager and selected hospitals’ Chief Executive Officers.

Data were collected at the selected healthcare facilities for a period of one month in June 2021. The interview guide comprised of one central question, which guided the interviews, namely “*what is your views about the control measures to reduce the risk of TB transmission in this facility?”* Participants were probed to elicit more information from them, and the probing questions were dependent on participants’ responses to the central question. The central question was developed in English and later translated to Tshivenda and Xitsonga. This was done before the pretest. A pretest was conducted to check the clarity of the central question as well as to identify possible flaws such as the researcher’s probing skills, which were improved after the pretest. Participants who were part of the pretest were not included in the main study, but their findings were included to avoid missing out on important information. Participants granted written permission to the use of voice recorder, which captured data as a backup to field notes, which were taken during the interviews. This was done to avoid missing important information. Each interview lasted for 30 to 45 min. Data saturation was reached at the 12th participant. However, the researcher went on with the interview by adding four (4) participants to make a total of sixteen (16) to ensure that indeed there was nothing new.

### 2.4. Data Analysis

Data from voice recordings were transcribed verbatim and translated into English [14]. Transcripts were sent back to the participants to ensure that what had been transcribed was the true reflection of what the participants said. This was done so that participants may have an opportunity to comment or make corrections to the data collected. Transcripts were coded by one independent contracted coder without the aid of software for thematic analysis and three themes and six sub-themes that were identified in advance guided the analysis.

### 2.5. Trustworthiness

All four measures to ensure trustworthiness were adhered to including credibility, dependability, conformability and transferability. Credibility was ensured by prolonged engagement and member checking. Experts were used to validate the methodology, which was further enhanced using an independent coder to ensure consistency, thereby enhancing dependability. Transferability was ensured through collection of detailed information and dense descriptions of the data. To ensure conformability, notes were retained in a secure location to allow for the creation of an acceptable trail and the determination of conclusions and interpretations.

## 3. Results

About three themes emerged and their subthemes from data analysis. These are the results from healthcare workers trained on TB management and who were available during the period of data collection. Of the sixteen (16) participants, eight (8) gave information that is related to theme one (1), four (4) participants’ responses were related to theme 2 and another four (4) were related to theme 3. However, an extra four (4) participants were added after data saturation to confirm that there was truly nothing new. Table 2 below shows themes and sub-themes that emerged from data analysis of raw data as presented.

### 3.1. Theme 1: Healthcare Workers’ Perceptions towards Administrative TB Control Measures

Healthcare workers in this study had positive perception regarding administrative control that seemed to be useful to reduce the development of new TB cases. The methods suggested by the healthcare workers were to reinforce and enhance triage and cough technique and to reduce delay in diagnostic measures.

#### 3.1.1. Triage

Triage was seen to be an effective measure to reduce new TB cases. This is because if properly done most patients in healthcare facilities can be free from infection, especially in overcrowded healthcare facilities. When infectious patients are not identified and separated immediately, many people may get infected. The study also revealed that it is very important to reduce the waiting period of the patients who are already diagnosed with TB in order to avoid further transmission of patients who are suspected but not yet diagnosed. These were some of the narratives from participants (coded “h” for healthcare workers and 1, 2, 3, etc., for number in the list):

*“………In this health facility due to high number of cases we decided to screen patients before we attended to them so that we can be able to identify suspects, isolate them from other patients and attended to them to avoid time spent in facility these has assisted in reducing the TB cases that we usually”*.

(h12)

This was supported by participant (h5) who said:

*“in this facility, we use to have more cases of TB in the whole local area, so we opted to benchmark on clinics that have low cases of TB so that we can reduce number of cases in our facility also. That is where we learnt that in the morning the help desk nurse tries to identify patients who are having cough and separate them from other patients so that they can be attended to first. This strategy helps because if ever a TB suspect test positive for TB, we would have done justice to other patients by preventing the transmission of the bacteria. Before applying this strategy, we use to have a new case every two weeks”*.

#### 3.1.2. Cough Etiquette

Health workers from this study highlighted cough etiquette as one of the best strategies to reduce the development of new TB cases. This study revealed that cough etiquette should be displayed on the walls of the healthcare facility. It is also important to demonstrate cough etiquette to the patients who are TB patients, those who are suspected and those who come for consultation so that they are able to do it and teach others that might not be doing it correctly. This cough etiquette is not only useful for TB prevention but is also useful to prevent the spread of COVID-19, which is a current pandemic that is also very infectious and killing lots of friend and relatives.

One healthcare worker (h9) said:

*“coughing on your elbow/tissue assist in preventing the spread of TB because if a healthcare worker is having latent TB and cough without following the coughing etiquette the healthcare worker can easily spread the bacteria to patients, he/she is attending to on daily basis. We attend to many patients a day; you can just imagine the number of patients we could infect. The same applies to patients. They need to be taught the coughing etiquette in order to prevent the transmission of TB pathogens to other patients or healthcare workers. A lot can occur in the waiting area if the coughing etiquette is not being followed.”*

This was emphasized by another healthcare worker (h2), who said:

*“coughing etiquette is the best for TB control. I mean we have people who cough on the palm of their hands and don’t wash their hands afterwards. What if the person is infected with TB? The bacteria could easily be spread during handshake. If our patients and healthcare workers follow the coughing etiquette, we can win this battle. Ever since I came to work in this clinic, it has never had zero (0) cases of TB.”*

#### 3.1.3. Reduction of Delay in Diagnosis

The study revealed that in most cases, patients are not diagnosed on time. This was seen to be a factor contributing to the increase in new TB cases. This is because delay in diagnosis leads to delay in the commencement of the TB treatment, which further increases the number of new TB cases as more people are infected unaware as even the one with TB positive results is not yet aware. Furthermore, the study noted that the delay is sometimes from the healthcare workers themselves when they delay in collecting the specimen to be analyzed for TB. It was also discovered that the laboratory personnel also release the TB results that are positive after a turnaround time of more than 48 h. Findings of these study revealed that if there is a delay in a patient being diagnosed with TB he or she may transmit the TB bacteria to other people because he/she will not know that he/she has an infectious condition, therefore he/she may continue to engage with friends and family as usual.

This is what some healthcare workers said:

*“During weekends and holidays there is no laboratory car which transport specimens from our clinic to the nearest laboratory, therefore we don’t collect specimens on weekends and holidays. Imagine if it’s a long weekend, these suspects can transmit the bacteria amongst their families and friends, because he/she will be waiting for diagnosis so that treatment can be commenced if necessary.”*

(h4)

*“We have patients who test negative but have all the signs of TB. These patients can be delayed in terms of diagnosis because he/she will have to go to hospital for chest x-ray which can delay also due to lack of transport money to hospital.”*

(h6)

Another healthcare worker (p15) said:

*“Since TB is mostly diagnosed with sputum, we have nurses who don’t like collecting sputum because they usually say it’s disgusting to see other people’s sputum. Even in this facility we have one nurse who will never collect sputum no matter the circumstances but will rather send patients who are eligible for sputum collection to a community healthcare worker. So, what happens if the community healthcare workers are not available in the facility? It ends up delaying the patient’s diagnoses. Delay in diagnosing can mean delay in commencement of treatment therefore allowing the patient to easily spread the TB bacteria.”*

Another healthcare worker (h13) said:

*“Sometimes these patients are difficult, a person will tell you I don’t have sputum before you even ask for it, just because he/she doesn’t want to produce sputum. So, what am I supposed to do in such kind of a situation except to just let the patient go?”*

### 3.2. Theme 2: Environmental Control Measures as Perceived by Healthcare Workers

Findings of this study reveal some environmental factors that reduce the spread of TB infections such as good ventilation and the use of germicidal ultraviolet lights. Participants indicated that these measures reduce the transmission of TB infection in the healthcare facilities and households if properly followed.

#### 3.2.1. Use of Germicidal Ultraviolet Lights

Study findings revealed that germicidal ultraviolet lights reduced the transmission of TB bacteria from one person to another as they have the power to trap the bacteria. Once the bacteria are trapped, it becomes difficult for a person to be infected. This is what participants (h13) indicated:

*“When the ultraviolet lights are functional, we have noticed a reduction in new TB cases, but it doesn’t last long because the lights are poorly maintained”*.

(h13)

Healthcare worker (h3) said:

*“Our health facilities have ultraviolet lights which assist in trapping the TB bacteria, but sometimes they become useless because of poor maintenance. It can take more than 5 years for them to be maintained. If only the responsible people were making sure that the lights are serviced every year, it can assist a lot in TB reduction.”*

#### 3.2.2. Natural Ventilation

Good ventilation was shown by this study to be a good practice that everyone must be involved in. When windows are opened, the bacteria receive enough space and roam in that room only as opposed to when the windows are closed; hence, good ventilation reduces the transmission of M tuberculosis. Healthcare facilities’ windows were seen to be open, which was good. The study emphasizes that the same practice should be carried out in our homes in order to reduce the transmission of TB bacteria, especially in overcrowded homes. 

A healthcare worker (h12) said:

*“Opening of windows in our consultation rooms can also assist in TB reduction. In our facility, the cleaner always open windows when she does her morning cleaning routine. It becomes a challenge during cold weathers because I have noticed that we close windows when busy attending to patient’s needs. These simply mean that since we don’t even have TB room, all patients who comes to the clinic on that day will be attended to in one room. These patients include the TB positive patients”*

*“……I know opening windows in the facility can allow airflow, but sometimes it becomes difficult due to infrastructure which is too small to accommodate all the clinic necessities. Some of the rooms don’t have enough space”*

(p6)

### 3.3. Theme 3: Perception of Healthcare Workers towards Respiratory Control Measures

Participants in this study revealed the importance of having knowledge regarding respiratory control measures. It is important for the healthcare workers to be acquainted with respiratory techniques so that they can properly assist patients when necessary. The knowledge includes when to use a respirator, how to remove it and when to change it. The researchers revealed that training of healthcare workers on infection control policy is essential as this may have a huge impact on reduction of new TB cases.

Participants in this study gave narratives and said: “*In our facility we do conduct in-service training on the use of respirators. We teach each other when and how we should wear a mask. We further teach each other’s how to dispose respiratory equipment. This knowledge assist in reducing the number of new TB cases because whenever I’m attending to a TB suspect or TB patient, I will know that I have to put respirators on”* (h7).

Healthcare worker (h11) said, *” being trained on the use of respirators made us realize that one of the reasons healthcare workers were getting TB infection was because they were not having essential; knowledge on how to dispose respirators that were used”.*

Another healthcare worker (h15) said, *“I think the government should make it a priority on training healthcare workers on use of respirators because I’m new here, but I have never received any training on such and I’m attending to TB patients daily. I believe these trainings can reduce the transmission rate of new TB cases”.*

## 4. Discussion

New TB cases are associated with the use of a triage system wherein a suspected TB individual is identified and given priority to be attended to so that the bacteria cannot be spread to the other individuals around them. Moreover, those who are in the queue will be protected by this practice. This was seen to be an effective way of reducing the new TB cases. Proper triage can also be effective if those who have already tested positive can be placed in one place and be separated from those who are still under investigation [14]. A study from Switzerland emphasizes the importance of avoiding contact when a person has symptoms. Similarly, another South African study by [15] indicated that TB suspects who are attended to immediately after it is suspected they are infected do not have many chances of transmitting the TB bacteria. Furthermore, it was also indicated that if only the healthcare workers were giving priority to TB suspects who are seriously ill and actively transmitting TB baccili, TB transmission could be reduced. Therefore, the healthcare workers need to have a clear understanding of the importance of triage, how it works and its benefit.

Cough etiquette was also identified by this study as a crucial factor to reduce new TB cases. However, health education to increase knowledge on what to do when coughing is vital. The information should be readily available, including the use of media, pamphlets and word of mouth. References [16,17] revealed that healthcare facilities that display information and give counselling to their patients are more likely to reduce TB transmission as compared to those without interventions. TB infection is non-discriminatory; it can affect any person at any given time, including young and old, rich and poor, literate and illiterate. Therefore, it is mandatory that healthcare workers practice cough etiquette as they can also infect patients. Cough etiquette should also go hand and glove with proper handwashing and other methods that are useful in the reduction of TB transmission.

The study revealed that new TB cases are increasing due to delays by healthcare workers. This was seen to be on the side of nurses, laboratory technicians and availability of resources. Nurses must always be conscious of the early diagnosis of suspected individuals. This must be fast tracked by carrying out the necessary investigation such as sputum or Mantoux test immediately when a person is identified as having symptoms. These delays become a barrier to early TB detection. Reference [18] discloses the barriers to early diagnosis and the health system was highlighted as one of the factors contributing to delays in TB diagnosis. This study also revealed delays caused by patient themselves, as they do not want to produce sputum when they are asked to. In such incidents, those patients are to be referred to the hospitals so that they can use other diagnostic methods such as X-ray. This becomes a problem again if the patient will not go to the hospital due to financial cost as they must use transport to the hospital [19]. Delays were also found to be caused by laboratories where results are released after 48 h. This may be due to increasing workload for the technician or lack of knowledge concerning the urgency of reporting the results to the relevant healthcare professionals. There must be a policy to be followed on how the results are to be followed, when and by who to enhance prevention and control of new infections This delay further increases the transmission of TB because those suspected will continue to infect other people unaware. Furthermore, Ref. [20] in their study concur with the study findings by indicating that the delay was mostly seen in TB lymphadenitis patients as compared to other types of TB. However, the findings of this study are contrary to the study conducted by [21], who confirmed that the results were released within 48 h.

Healthcare workers in this study perceived the use of germicidal ultraviolet as being useful in the reduction of new TB cases. However, the study further discovered that most of those germicidal ultraviolet lights were out of order and remained some years without being repaired. This demonstrated ignorance on the part of healthcare facility managers as the managers are expected to ensure that all equipment is in good working order. Reference [22] reported that the use of germicidal ultraviolet lights reduces the rate of transmission of bacteria in healthcare facility rooms. Ref. [23] indicated that use of ultraviolet light kills the microorganisms that are airborne. Low-pressure mercury vapor lamps emit UVC. It was further indicated that upper room germicidal ultraviolet systems are recommended to reduce M tuberculosis for healthcare workers and any person attending the health facility. Therefore, the healthcare managers should consider the use of germicidal ultraviolet lights for the benefits of both the patients and the healthcare workers as it cleanses and purifies air using an air cleaning method [24]. It must always be remembered that no one is immune from TB infection. Hence, prevention, early diagnosis and treatment are of vital importance.

Good ventilation is a basic way of preventing new TB cases, especially in overcrowded places. Findings of this study revealed challenges related to infrastructure that is too small to accommodate all patients. Moreover, due to lack of space and TB rooms, all patients are kept in one room irrespective of what they are suffering from. Once a person is staying in a place where there is no proper or adequate ventilation, the chance of contaminating TB infections is very high if there are TB positive cases. The findings of the study are similar to what [25] recommended. It was said that ventilation systems including natural mixed-mode, mechanical ventilation and re-circulated air though HEPA filters are recommended for healthcare workers/person attending healthcare facilities. However, Ref. [26] is of the opinion that assessment concerning the sufficiency of proper ventilation is carried out through monitoring the number of air changes per hour (ACH).

The study revealed the need to train healthcare workers on the respiratory control measures, especially new healthcare workers. Thorough orientation and induction for new staff members is necessary for them not to be infected with TB. They need to be trained on the proper disposal of the used respirators. Even if there may be few untrained personnel, training is still very important as the number of people that might be infected can be greater if the proper handling of respirators is not adhered to. The study results are supported by [13], who revealed that the majority of healthcare workers under study were not aware of the respiratory techniques that they have to follow in the ward which resulted in transmission of the bacteria. Ref. [27] revealed that by providing disposable N95 respirators and fit test training or education we can reduce the new cases of TB.

The implications for this study in clinical practice is that the identified challenges such as closing windows during winter, UVGI lights that are non-functional and taking too long to be fixed, no specimen collection during weekends and holidays, thereby delaying TB diagnosis and lack of skills concerning how to use respirators and cough etiquette at clinics in Collins Chabane Municipality need to be addressed by Vhembe district TB control programme staff if TB is to be eradicated by 2030.

## 5. Limitations

This study was conducted in only one municipality out of five in the Vhembe district, South Africa. Thus, the results do not give a district, province or country picture of healthcare workers’ perceptions concerning the implementation of TB preventative measures. Further research needs to consider other municipalities and districts to inform Limpopo policy interventions.

## 6. Conclusions

Tuberculosis administrative, environmental and respiratory control measures are well understood by health workers. However, there are still challenges with implementation of some measures, such as closing windows during winter, UVGI lights that are non-functional and taking too long to be fixed, no specimen collection during weekends and holidays, thereby delaying TB diagnosis, and lack of skills concerning how to use respirators and cough etiquette at clinics in Collins Chabane Municipality. The Vhembe district TB control programme should intensify training and continue monitoring correct implementation, giving needed support.

## Figures and Tables

**Table 1:** Biographical information of healthcare workers.

Participants	Gender	Age	Rank	Level of Education	Years of Experience
1	Female	45	Professional nurse	Degree	16
2	Female	52	Professional nurse	Degree	21
3	Female	41	Professional nurse	Diploma	15
4	Female	30	Professional nurse	Degree	06
5	Female	38	Enrolled nurse	Diploma	12
6	Female	27	Professional nurse	Degree	04
7	Female	54	Professional nurse	Diploma	24
8	Female	36	Enrolled nurse	Diploma	13
9	Male	40	Enrolled nurse	Diploma	15
10	Male	25	Professional nurse	Degree	04
11	Male	30	Professional nurse	Degree	09
12	Male	32	Professional nurse	Degree	11
13	Male	23	Professional nurse	Degree	02
14	Male	28	Professional nurse	Degree	07
15	Male	30	Professional nurse	Degree	09
16	Male	32	Professional nurse	Degree	11

**Table 2:** Summary of findings from data analysis.

Themes	Sub-Themes
Administrative control	Triage Cough etiquette Reduction in delay in diagnostic measures
Environmental control	Use of germicidal ultraviolet Good ventilation
Respiratory control	Training of healthcare workers on respiratory measures.

## Data Availability

Data available upon request.
